# Glandular amputation by strangulating tied suture: a case report of late-onset complication in the Plastibell circumcision technique

**DOI:** 10.1186/s12887-019-1554-z

**Published:** 2019-06-01

**Authors:** Jalil Hosseini, Saeid Haghdani, Nima Narimani

**Affiliations:** 1 Men’s health and reproductive health research center, Shahid Beheshti University of Medical Sciences, Tehran, Iran; 2 Department of Urology, Hasheminejad Kidney Center (HKC), Iran University of Medical Sciences (IUMS), Tehran, Iran

**Keywords:** Circumcision, Plastibell, Complication

## Abstract

**Background:**

Circumcision is considered to be a procedure with minimal morbidity but may be associated with catastrophic complications in inexpert hands.

**Case presentation:**

We presented a 9-year-old boy with a past medical history of circumcision at the age of one year with Plastibell clamp who was referred with severe chronic penile injury due to neglected plastibell string. After string removal under a loupe magnification (4×), we saw a deep circular injury at distal penile shaft which led to painless glandular autoamputation 45 days later. The patient was managed conservatively with daily urethral self-dilation until future reconstructive surgery.

**Conclusion:**

This complication emphasized the importance of the follow-up visit by a physician for any probable string remnant.

## Background

Circumcision is the most common pediatric surgery worldwide, and performed due to cultural, religious and medical reasons [1]. It may be beneficial in reducing urinary tract infection, phimosis, balanitis, sexually transmitted infection and genital cancer [2]. Circumcision in newborn and in infancy has fewer complications due to their impressive healing capability and simpler techniques [3]. Complications of circumcision are usually minor and benign in most of the cases. However, there are reports about rare major complications with considerable morbidity, in which the treatment is challenging. In this case report, we present a 9-year-old boy with chronic penile injury due to a missed Plastibell string that finally lead to glandular autoamputation within one month after string removal.

## Case presentation

**Fig. 1:**
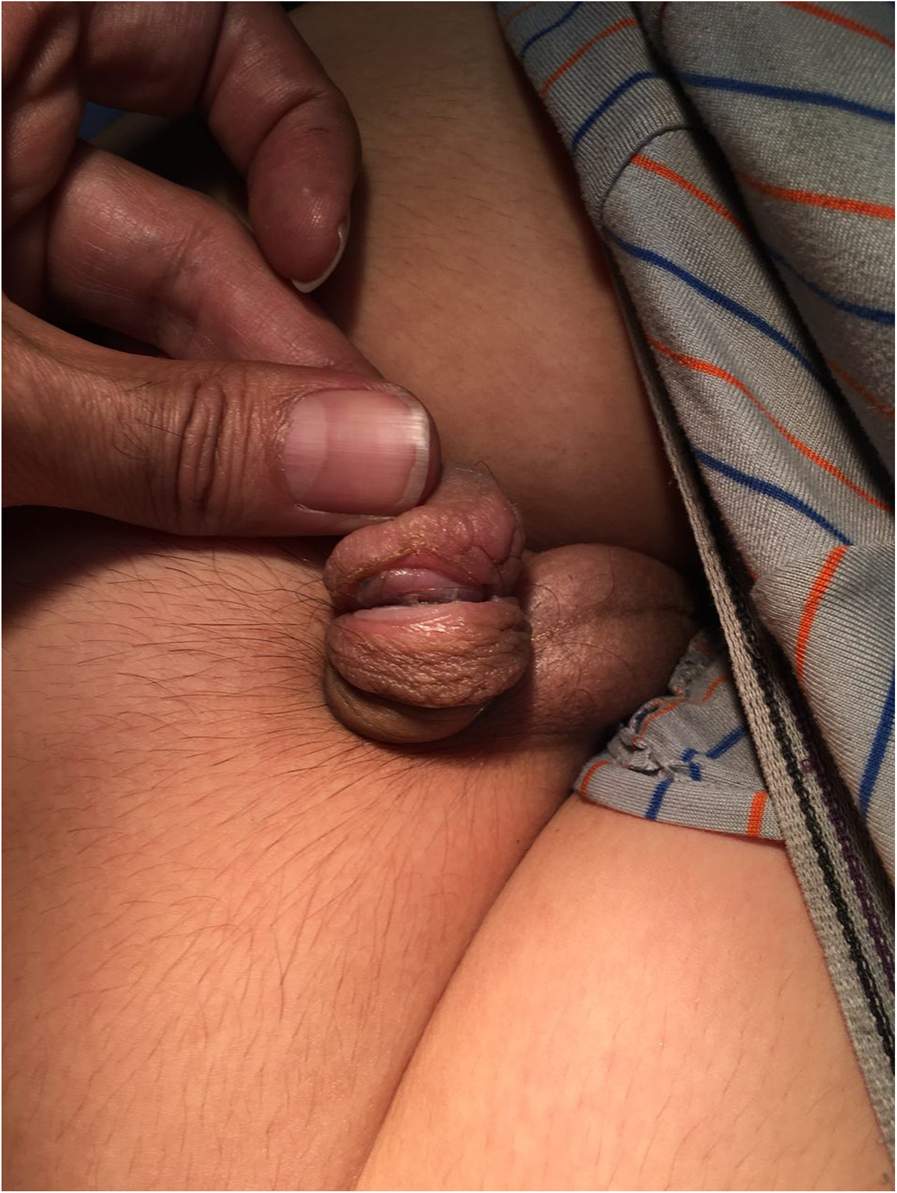
A circular sharp cutting in the distal penile shaft, where the tied string usually is placed in plastibell circumcision

**Fig. 2:**
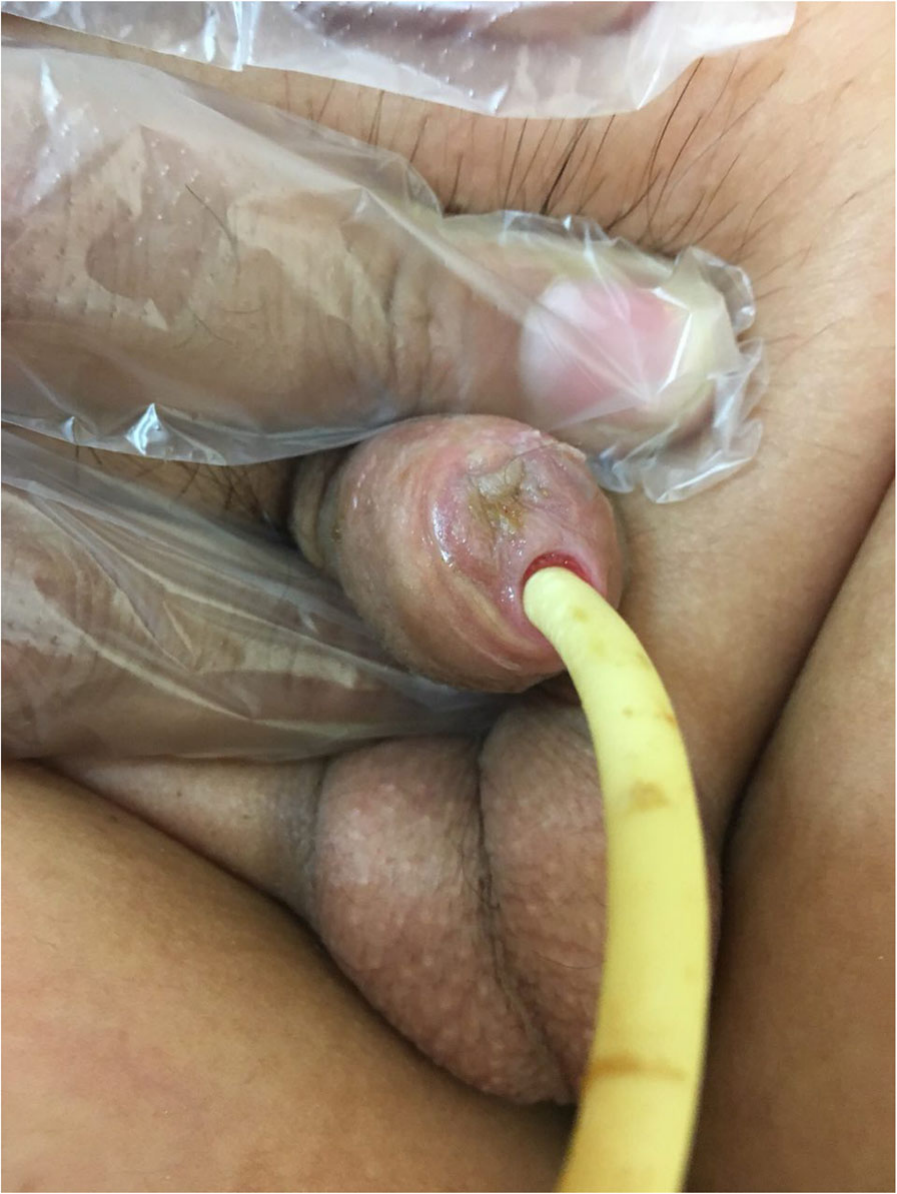
The penis appearance after auto-amputated glans removal

A 9-year-old boy was brought to the urology clinic due to severe decreased urinary force and caliber since one month earlier. He has mentioned dysuria without other irritative lower urinary tract symptoms, intermittent hematuria, or downward urination. In past medical history, he was circumcised at the age of one year. In the physical examination, the glans, meatus and penile shaft seemed to be normal initially. Nevertheless, with precise inspection, a deep circular sharp cut in peno-glanular junction, was detected (Fig. 1). In the operating room, under local anesthesia and loupe magnification(X4), we detected a neglected Plastibell remnant string, which was removed with an eye scissor, and a 6 French Foley catheter was inserted in the urethra for two weeks. The patient was referred to a tertiary urethral and penile reconstructive center. Unfortunately, the penile glans auto amputation spontaneously occured after 45 days during the daily activity with minimal bleeding (Fig. 2). Foley catheter was again inserted for 2 weeks and a watch-full waiting approach for three months was recommended. He is now managed conservatively with daily urethral self-dilation and is candidate for future reconstructive surgery.

## Discussion and conclusions

Male circumcision is one of the most common surgical procedures worldwide, and is commonly performed in newborn and during infancy. The clamp-based techniques such as Gomco, Plastibell and Mogen clamp are the most popular circumcision methods in newborns [4]. The Plastibell technique was introduced in the 1950s and is the most common method for circumcision in our country [5]. The Plastibell consists of a plastic ring which is placed between the foreskin and the glans (to protect the glans from iatrogenic injury) and a string is used to clamp the foreskin into the groove in the ring. While this method is recommended for children under one year, open technique is used in a wide age range. However there are some reports about safety and feasibility of plastibell in older children [6]. The complication rate for circumcision varies widely from 0 to 16% [7]. They are categorized as early and late complications. While early complications mainly consist of bleeding, infection and unsatisfactory cosmetic results, the late ones are classified into the minor and major forms. Minor complications like meatal stenosis and penile adhesion can be managed easily, whereas the treatment of major complications like extensive penile skin lost, urethrocutaneous fistula and penile amputation is quite challenging [8].

Glandular necrosis and amputation were previously reported as one of the most severe complications and were mostly due to use of electrocautery, sharp iatrogenic injury [9, 10] or the proximal migration of plastibell instrument [10]. To the best of our knowledge, this is the first case of complete glans autoamputation due to neglected remained plastibell hemostatic string, which occurred several years after circumcision. Although the parents are usually fully informed to bring back the child in the case of delayed plastibell falling off [11], a routine follow up visit (by an expert) in regards to string remnant has not been emphasized till now. We suggest that this exam should be performed (by the physician and not the parents) soon after the ring fell off, to prevent such catastrophic consequences.

## Data Availability

The datasets used during the current study are available from the corresponding author. The data are only images which are collected by corresponding author and permission for sharing is obtained from patient’s parent.
